# Survival Outcomes Associated With Cytoreductive Nephrectomy in Patients With Metastatic Clear Cell Renal Cell Carcinoma

**DOI:** 10.1001/jamanetworkopen.2022.12347

**Published:** 2022-05-16

**Authors:** Nicholas H. Chakiryan, L. Robert Gore, Richard R. Reich, Rodney L. Dunn, Da David Jiang, Kyle A. Gillis, Elizabeth Green, Ali Hajiran, Lee Hugar, Logan Zemp, Jingsong Zhang, Rohit K. Jain, Jad Chahoud, Philippe E. Spiess, Brandon J. Manley, Wade J. Sexton, Brent K. Hollenbeck, Scott M. Gilbert

**Affiliations:** 1 Department of Genitourinary Oncology, H. Lee Moffitt Cancer Center, Tampa, Florida; 2 Department of Biostatistics and Bioinformatics, H. Lee Moffitt Cancer Center, Tampa, Florida; 3 Department of Urology, University of Michigan Medical School, Ann Arbor; 4 Department of Urology, Beth Israel Deaconess Medical Center, Boston, Massachusetts; 5 Department of Urology, University of Iowa Hospitals & Clinics, Iowa City

## Abstract

**Importance:**

Level I evidence has failed to demonstrate an overall survival (OS) advantage for cytoreductive nephrectomy in patients with metastatic clear cell renal cell carcinoma (ccRCC) in the modern era, which is at odds with observational studies reporting a marked OS benefit associated with these operations. These observational studies were not designed to adjust for unmeasured confounding.

**Objective:**

To assess whether cytoreductive nephrectomy is associated with improved OS in patients with metastatic ccRCC.

**Design, Setting, and Participants:**

This cohort study identified patients with metastatic ccRCC in the National Cancer Database from January 1, 2006, to December 31, 2016, who received systemic targeted therapy. The analysis was finalized on July 23, 2021.

**Exposures:**

Receipt of cytoreductive nephrectomy.

**Main Outcomes and Measures:**

The primary outcome was OS from the date of diagnosis to death or censoring at last follow-up. Distance from the patients’ zip code of residence to the treating facility was identified as a valid instrument and was used in a 2-stage residual inclusion instrumental variable analysis. Conventional adjustments for selection bias, multivariable Cox proportional hazards regression, and propensity score matching were performed for comparison. Measured covariates adjusted for in all analyses included age, sex, race, Charlson-Deyo score, facility type, year of diagnosis, clinical T stage, and clinical N stage.

**Results:**

The final study population included 12 766 patients (median age, 63 years; IQR, 56-70 years; 8744 [68%] male; 11 206 [88%] White). Cytoreductive nephrectomy was performed in 5005 patients (39%). Conventional adjustments for selection bias demonstrated a significant OS benefit associated with cytoreductive nephrectomy (multivariable Cox proportional hazards regression: hazard ratio [HR], 0.49; 95% CI, 0.47-0.51; propensity score matching: HR, 0.48; 95% CI, 0.46-0.50). Instrumental variable estimates did not demonstrate an association between cytoreductive nephrectomy and OS (HR, 0.92; 95% CI, 0.78-1.09).

**Conclusions and Relevance:**

Instrumental variable analysis did not demonstrate a survival advantage associated with cytoreductive nephrectomy for patients with metastatic ccRCC. This discrepancy likely reflects the fact that surgical indication for cytoreductive nephrectomy is primarily driven by factors that are not commonly measured or available in observational data sets.

## Key Points

**Question:**

Is cytoreductive nephrectomy associated with improved overall survival for patients with metastatic clear cell renal cell carcinoma?

**Findings:**

In this cohort study of 12 766 patients, receipt of a cytoreductive nephrectomy was not associated with improved overall survival using instrumental variable analysis to adjust for bias due to unmeasured confounding. Methods that do not adjust for this bias would have suggested a substantial overall survival benefit.

**Meaning:**

Consistent with contemporary Level I evidence, instrumental variable analysis demonstrated that cytoreductive nephrectomy was not associated with improved overall survival for patients with metastatic clear cell renal cell carcinoma.

## Teaser

This cohort study of patients with metastatic clear cell renal cell carcinoma assesses whether cytoreductive nephrectomy is associated with improved overall survival.

## Introduction

For decades, cytoreductive nephrectomy has been a clinical standard for patients with metastatic clear cell renal cell carcinoma (ccRCC) who are surgical candidates and do not harbor poor-risk disease.^1^ This practice was initially grounded by randomized clinical trials in the cytokine era demonstrating improved overall survival (OS) associated with the receipt of cytoreductive nephrectomy plus interferon alfa compared with patients who received interferon alfa alone.^2,3,4^ Shortly after these trials were conducted, the discovery of tyrosine kinase–inhibiting targeted therapies markedly shifted first-line systemic management away from cytokine therapy.^5^ Since this paradigm shift, several large observational studies^6,7,8^ have reported that cytoreductive nephrectomy continued to provide a substantial OS benefit during the postcytokine era.

Published in 2018, the CARMENA (Cancer du Rein Metastatique Nephrectomie et Antiangiogéniques) trial^9^ randomized patients with intermediate- or poor-risk metastatic ccRCC to initial cytoreductive nephrectomy followed by sunitinib vs sunitinib alone and found that sunitinib alone was a noninferior treatment strategy for OS (hazard ratio [HR], 0.89; 95% CI, 0.71-1.10; noninferiority margin, 1.20). Although this trial has several important limitations, it remains the only available high-level evidence addressing the efficacy of cytoreductive nephrectomy in the postcytokine era. Shortly after CARMENA, the SURTIME (Immediate Surgery or Surgery After Sunitinib Malate in Treating Patients With Metastatic Kidney Cancer) randomized clinical trial^10^ reported that patients with metastatic ccRCC who received both cytoreductive nephrectomy and sunitinib had significantly improved OS when sunitinib was administered upfront. The SURTIME results further suggest that upfront cytoreductive nephrectomy is a suboptimal treatment strategy but do not address whether delayed nephrectomy is preferable to no nephrectomy at all. Nevertheless, cytoreductive nephrectomy continues to be practiced as a therapeutic procedure for the management of metastatic ccRCC, largely based on observational data indicating a significant survival benefit.^6,7,8,11,12^

These observational studies^6,7,8^ did not account for selection bias related to unmeasured confounding by surgical indication, and as such their results may not accurately reflect the effectiveness of the intervention. Conventional survival analyses using observational data can only adjust for variables that are available in the data source.^13^ In the case of cytoreductive nephrectomy, the decision to proceed with surgery in an otherwise qualified patient is nuanced and based on a multitude of patient- and disease-specific factors, such as patient frailty, surgical complexity, and the site and volume of metastases. These variables are generally absent from observational data sources. Advanced statistical approaches, such as instrumental variable analysis, can be used to leverage natural variation, mimic randomization, and account for unmeasured confounding but are associated with specific conditions and assumptions, require large patient samples, and are therefore infrequently used in observational analyses.^13,14,15,16,17^ The primary objectives of this analysis were to assess the effect of cytoreductive nephrectomy on OS for patients with metastatic ccRCC using instrumental variable analysis to adjust for unmeasured confounding and to compare these results with those generated by conventional adjustments for selection bias.

## Methods

### Study Population and Data Source

Cases of ccRCC were identified and abstracted from the National Cancer Database (NCDB) from January 1, 2006, to December 31, 2016. This study was conducted using deidentified patient data, and as such was deemed exempt by the H. Lee Moffitt Cancer Center and Research Institute’s institutional review board. This study followed the Strengthening the Reporting of Observational Studies in Epidemiology (STROBE) reporting guideline.^18^

The NCDB includes more than 70% of incident cancer cases diagnosed in the US, which are reported by member facilities of the Commission on Cancer. Trained data abstractors collect and submit data to the NCDB using standardized coding definitions as specified in the most recent Commission on Cancer Facility Oncology Registry Data Standards (FORDS) guideline.^19^ Data on race and ethnicity were collected to adjust for potential confounding caused by these factors. Race and ethnicity data are also classified and reported according to the FORDS guideline.

The year 2006 was chosen as the earliest date for the analysis because this was the first year of US Food and Drug Administration approval of sunitinib for the treatment of advanced RCC.^20^ Criteria for case inclusion were clinical stage IV metastatic ccRCC at diagnosis, age of 18 to 100 years, availability of complete staging and demographic data, and receipt of targeted therapy as first-line treatment. Cases were excluded if the NCDB codes indicated that the patient was treated on an experimental or blinded clinical trial protocol. Cases were excluded if data were missing on distance from the patient’s residence to the treating facility. The International Metastatic Renal Cell Carcinoma Database risk score is not available in the NCDB, and available data points are not adequate to calculate this score directly.

### Variables and Definitions

Consistent with previously peer-reviewed and published NCDB studies^8,21,22,23^ regarding targeted therapy use in RCC, targeted therapy was defined as the receipt of single or multiagent systemic chemotherapy. Age was defined as the age at initial diagnosis. Comorbidities were measured according to the Charlson-Deyo method and scored as discrete count categories (0, 1, 2, or ≥3) per NCDB reporting standards.^24,25^ Distance to the treating facility was defined as the great-circle distance, in miles, from the zip code centroid of the patient’s residence to the geocode corresponding to the street address of the treating facility. Overall survival was measured from the date of initial diagnosis to the date of death or censorship at last follow-up.

### Statistical Analysis

Patients were stratified based on cytoreductive nephrectomy status. Baseline patient and tumor characteristics were abstracted from the data set and compared between groups. Wilcoxon rank sum testing was used to compare continuous variables, and the χ^2^ test of independence was used to compare categorical variables.

To apply a conventional adjustment for selection bias attributable to measured confounding, a multivariable Cox proportional hazards regression was performed on the overall cohort using the following covariates: age, sex, race, Charlson-Deyo score, facility type, year of diagnosis, cT stage, cN stage, and cytoreductive nephrectomy status. Kaplan-Meier estimates were used to generate survival functions.

Another conventional adjustment for measured confounding was applied, using 1:1 nearest-neighbor propensity score matching, without replacement, with a caliper width of 0.2 SDs of the propensity score distribution, as previously described.^26,27^ The baseline variables used to formulate propensity scores included age, sex, race, Charlson-Deyo score, facility type, year of diagnosis, cT stage, and cN stage. Univariable analysis was repeated in the postmatching cohorts to assess for balance between groups. The a priori design for the propensity score–matched survival analysis was set to include any variables from the post matching univariable analysis with *P* values <.10 as covariates in a multivariable Cox proportional hazards regression for OS, including cytoreductive nephrectomy status as the exposure variable. Kaplan-Meier estimates were used to generate survival functions among the patients in the matched cohort.

Instrumental variable analysis is a method initially developed for econometrics that relies on an instrumental variable that is associated with the exposure variable and is unrelated to the outcome of interest except through its effect on the exposure.^14,15,28,29^ With adjustment for measured confounding variables, instrumental variable estimates compare the marginal patients, meaning those whose exposure status was driven primarily by the instrumental variable, resulting in pseudorandomization and mitigating the effect of selection bias through unmeasured confounding.^14,15,28,29^ This method reflects clinical trial results more closely than conventional adjustments for selection bias.^30^

Distance from the patient’s residence to the treating facility was assessed as a candidate instrumental variable. Distance to facility was assessed as a continuous variable, correcting for positive skewness with a natural logarithmic transformation. Travel distance is one of the most commonly used instrumental variables,^13^ including the landmark study by Card^31^ associating education level and future earnings. A multivariable logistic regression analysis was performed for cytoreductive nephrectomy status (the first-stage model), including age, sex, race, Charlson-Deyo score, facility type, year of diagnosis, cT stage, and cN stage as measured confounders, in addition to the candidate instrument: distance to facility. The distance to facility variable was then removed from the list of covariates, creating a second regression model, and the fit of the 2 regression models was compared using the *F* statistic, as previously described.^32^ Commonly accepted convention dictates that if the *F* statistic of this comparison is greater than 10, then the instrument is considered relevant.^14^ In this case, the *F* statistic was 33.3 (*P* < .001). Multivariable Cox proportional hazards regression for OS was then used, including the same covariates as the first-stage model, to confirm that the distance to facility was not independently associated with OS. The *F* statistic was determined for 2 multivariable Cox proportional hazards regressions: with and without the distance to facility variable. Visualization of the log-log plot for this regression confirmed that the proportional hazards assumption was not violated.

Once a valid and relevant instrument was identified, 2-stage residual inclusion instrumental variable estimates were determined, as previously described.^16,17,33^ For 2-stage residual inclusion methods, the first-stage model is as described in the previous paragrach, and the second-stage model is a multivariable time-to-event regression, including the residual from the first-stage model in addition to the measured confounding predictors and the exposure variable. The inclusion of the first-stage residual significantly decreases bias in nonlinear models compared with 2-stage predictor substitution methods.^16^ The SEs were calculated by stacking all estimating equations and applying a sandwich estimate, as previously described.^33,34^

A subgroup analysis was conducted for each of the 3 methods (multivariable Cox proportional hazards regression, propensity score matching, and instrumental variable analysis) to test whether the cytoreductive nephrectomy sequence with targeted therapy administration (upfront vs delayed cytoreductive nephrectomy) was associated with OS.

All analyses maintained the standard definition of statistical significance as a 2-tailed α = .05. All statistical analyses and data visualization were performed using R, version 4.0.2 (R Foundation for Statistical Computing). Instrumental variable validity testing and 2-stage residual inclusion analysis were performed using the lmtest and ivtools packages, propensity matching was performed using the MatchIt package, survival analyses were performed using the survival and survminer packages, tables were generated using the gtsummary package, and forest plots were generated using the forestplot package. The analysis was finalized on July 23, 2021.

## Results

### Study Population

**Figure 1.**
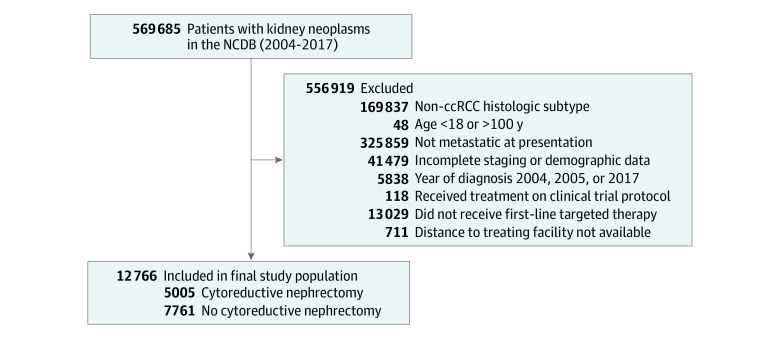
Selection of the Study Population ccRCC indicates clear cell renal cell carcinoma; NCDB, National Cancer Database.

We identified 569 685 patients in the NCDB with a kidney or renal pelvis neoplasm, and after applying the inclusion and exclusion criteria, the final study population included 12 766 patients (median age, 63 years; IQR, 56-70 years; 8744 [68%] male; 1033 [8%] Black; 11 206 [88%] White; 527 [4%] other race or ethnicity) (Figure 1). Cytoreductive nephrectomy was performed in 5005 patients (39%). Univariable analysis demonstrated that patients who underwent a cytoreductive nephrectomy were younger (61 vs 65 years), were more likely to be White (90% vs 86%), had lower Charlson-Deyo scores (Charlson-Deyo score of 0: 73% vs 69%), and were more likely to be treated at an academic facility (43% vs 36%) (Table 1).

**Table 1.** Baseline Patient and Tumor Characteristics^a^

Characteristic	All patients (N = 12 766)	No cytoreductive nephrectomy (n = 7761)	Cytoreductive nephrectomy (n = 5005)	*P* value^b^
Age, median (IQR), y	63 (56-70)	65 (57-72)	61 (54-67)	<.001
Sex				
Male	8744 (68)	5279 (68)	3465 (69)	.20
Female	4022 (32)	2482 (32)	1540 (31)	
Race				
Black	1033 (8)	749 (10)	284 (6)	<.001
White	11 206 (88)	6700 (86)	4506 (90)	
Other^c^	527 (4)	312 (4)	215 (4)	
Charlson-Deyo score				
0	8999 (70)	5356 (69)	3643 (73)	<.001
1	2679 (21)	1649 (21)	1030 (21)	
2	740 (6)	516 (7)	224 (4)	
≥3	348 (4)	240 (3)	108 (2)	
Facility type				
Academic	4913 (38)	2763 (36)	2150 (43)	<.001
Nonacademic	7853 (62)	4998 (64)	2855 (57)	
Year of diagnosis, median (IQR)	2012 (2010-2014)	2012 (2010-2014)	2012 (2010-2014)	.11
cT				
cT1	3182 (25)	2295 (30)	887 (18)	<.001
cT2	3934 (31)	2213 (29)	1721 (34)	
cT3	4150 (33)	2101 (27)	2049 (41)	
cT4	1500 (12)	1152 (15)	348 (7)	
cN				
cN0	7945 (62)	4366 (56)	3579 (72)	<.001
cN+	4821 (38)	3395 (44)	1426 (28)	

^a^ Data are presented as number (percentage) of patients unless otherwise indicated.

^b^ Statistical tests performed were the Wilcoxon rank sum test and the χ^2^ test of independence.

^c^ Other race or ethnicity includes American Indian, Aleutian or Eskimo, Chinese, Japanese, Filipino, Hawaiian, Korean, Vietnamese, Laotian, Hmong, Kampuchean (including Khmer and Cambodian), Thai, Asian Indian or Pakistani not otherwise specified, Asian Indian, Pakistani, Micronesian, Chamorran, Guamanian, Polynesian, Tahitian, Samoan, Tongan, Melanesian, Fiji Islander, New Guinean, and Pacific Islander.

Multivariable Cox proportional hazards regression analysis of the overall cohort identified improved OS associated with cytoreductive nephrectomy (HR, 0.49; 95% CI, 0.47-0.51) (Table 2). Unadjusted Kaplan-Meier estimates demonstrated a significant OS benefit associated with cytoreductive nephrectomy (eFigure 1A in the Supplement).

**Table 2.** Multivariable Cox Proportional Hazards Regression for Overall Survival (N = 12 766)

Characteristic	HR (95% CI)	*P* value
Age (per year)	1.00 (1.00-1.01)	<.001
Sex		
Male	1 [Reference]	NA
Female	1.04 (0.99-1.08)	.09
Race		
Black	1.10 (1.03-1.18)	.007
White	1 [Reference]	NA
Other^a^	1.00 (0.90-1.10)	>.99
Charlson-Deyo score		
0	1 [Reference]	NA
1	1.06 (1.01-1.11)	.02
2	1.07 (0.99-1.16)	.10
≥3	1.2 (1.07-1.36)	.002
Facility type		
Academic	1 [Reference]	NA
Nonacademic	1.17 (1.12-1.21)	<.001
Year of diagnosis (per year)	0.96 (0.95-0.97)	<.001
cT		
cT1	1 [Reference]	NA
cT2	1.19 (1.12-1.25)	<.001
cT3	1.20 (1.14-1.26)	<.001
cT4	1.29 (1.21-1.38)	<.001
cN		
cN0	1 [Reference]	NA
cN+	1.42 (1.37-1.48)	<.001
Cytoreductive nephrectomy		
No	1 [Reference]	NA
Yes	0.49 (0.47-0.51)	<.001

Abbreviations: NA, not applicable; HR, hazard ratio.

^a^ Other race or ethnicity includes American Indian, Aleutian or Eskimo, Chinese, Japanese, Filipino, Hawaiian, Korean, Vietnamese, Laotian, Hmong, Kampuchean (including Khmer and Cambodian), Thai, Asian Indian or Pakistani not otherwise specified, Asian Indian, Pakistani, Micronesian, Chamorran, Guamanian, Polynesian, Tahitian, Samoan, Tongan, Melanesian, Fiji Islander, New Guinean, and Pacific Islander.

Nearest neighbor propensity score caliper matching resulted in a cohort of 9322 patients: 2 equally sized groups of 4661 patients according to cytoreductive nephrectomy status (eFigure 2 in the Supplement). Univariable analysis demonstrated statistically significant findings for the following measured confounders: age (median [IQR] age: 62 [54-69] years in the no cytoreductive nephrectomy group and 61 [54-68] years in the cytoreductive nephrectomy group; *P* = .06), cT stage (cT1: 959 [21%] in the no cytoreductive nephrectomy group and 881 [19%] in the cytoreductive nephrectomy group: cT2: 1630 [35%] in the no cytoreductive nephrectomy group and 1606 [34%] in the cytoreductive nephrectomy group; cT3: 1699 [36%] in the no cytoreductive nephrectomy group and 1826 [39%] in the cytoreductive nephrectomy group; cT4: 373 [8.0%] in the no cytoreductive nephrectomy group and 348 [7.5%] in the cytoreductive nephrectomy group; *P* = .03); and cN stage (cN0: 3123 [67%] in the no cytoreductive nephrectomy group and 3249 [70%] in the cytoreductive nephrectomy group; cN+: 1538 [33%] in the no cytoreductive nephrectomy group and 1412 [30%] in the cytoreductive nephrectomy group; *P* = .005) (eTable 1 in the Supplement). Per the a priori analysis plan, these variables were included in a multivariable Cox proportional hazards regression with cytoreductive nephrectomy status as the exposure variable. This regression demonstrated significantly improved OS associated with receipt of cytoreductive nephrectomy (HR, 0.48; 95% CI, 0.46-0.50) (eTable 2 in the Supplement). With the use of the matched cohort, Kaplan-Meier plots of the prematching and postmatching cohorts can be found in eFigure 1B in the Supplement.

A directed acyclic graph depicting the structure and underlying assumptions of instrumental variable estimates is provided in eFigure 3A in the Supplement. Distance to facility was determined to be a relevant instrumental variable as demonstrated in the first-stage model by an *F* statistic of 33.3 (*P* < .001), with an increasing proportion of patients undergoing cytoreductive nephrectomy receipt as distance to facility increased (eFigure 3B and 3C in the Supplement). Distance to facility was not associated with OS, as demonstrated by a multivariable Cox proportional hazards regression *F* statistic of 1.7 (*P* = .20) (eFigure 3 in the Supplement). Baseline patient and tumor characteristics stratified by distance to facility tertiles are available in eTable 3 in the Supplement.

The 2-stage residual inclusion instrumental variable estimate did not demonstrate an association between cytoreductive nephrectomy status and OS (HR, 0.92; 95% CI, 0.78-1.09; after inversion of the β-coefficient signs, HR, 1.09; 95% CI, 0.92-1.28) (Table 3). Covariates associated with worse OS included older age (HR, 1.01; 95% CI, 1.00-1.01), Black race (HR, 1.12; 95% CI, 1.02-1.23), treatment at a nonacademic facility (HR, 1.11; 95% CI, 1.05-1.17), cT4 primary tumor stage (HR, 1.24; 95% CI, 1.12-1.37), and cN+ nodal stage (HR, 1.53; 95% CI, 1.44-1.62). Median follow-up time for patients who were alive at last contact was 36.0 months (IQR, 22.3-56.7 months).

**Table 3.** Two-Stage Residual Inclusion Estimate for Overall Survival (N = 12 766)

Variable	HR (95%CI)	*P* value
First-stage residual [X - Ê(X|L,Z)]	0.98 (0.93-1.04)	.57
Age (per year)	1.01 (1.00-1.01)	<.001
Sex		
Male	1 [Reference]	NA
Female	1.05 (0.99-1.11)	.14
Race		
Black	1.12 (1.02-1.23)	.02
White	1 [Reference]	NA
Other^a^	0.96 (0.82-1.11)	.56
Charlson-Deyo score		
0	1 [Reference]	NA
1	1.06 (0.99-1.13)	.11
2	1.06 (0.95-1.19)	.30
≥3	1.02 (0.87-1.19)	.80
Facility type		
Academic	1 [Reference]	NA
Nonacademic	1.11 (1.05-1.17)	<.001
Year of diagnosis (per year)	0.94 (0.92-0.96)	<.001
cT		
cT1	1 [Reference]	NA
cT2	1.08 (1.00-1.16)	.06
cT3	1.03 (0.96-1.10)	.45
cT4	1.24 (1.12-1.37)	<.001
cN		
cN0	1 [Reference]	NA
cN+	1.53 (1.44-1.62)	<.001
Cytoreductive nephrectomy		
No	1 [Reference]	NA
Yes	0.92 (0.78-1.09)	.32

Abbreviations: NA, not applicable; HR, hazard ratio.

^a^ Other race or ethnicity includes American Indian, Aleutian or Eskimo, Chinese, Japanese, Filipino, Hawaiian, Korean, Vietnamese, Laotian, Hmong, Kampuchean (including Khmer and Cambodian), Thai, Asian Indian or Pakistani not otherwise specified, Asian Indian, Pakistani, Micronesian, Chamorran, Guamanian, Polynesian, Tahitian, Samoan, Tongan, Melanesian, Fiji Islander, New Guinean, and Pacific Islander.

**Figure 2.**
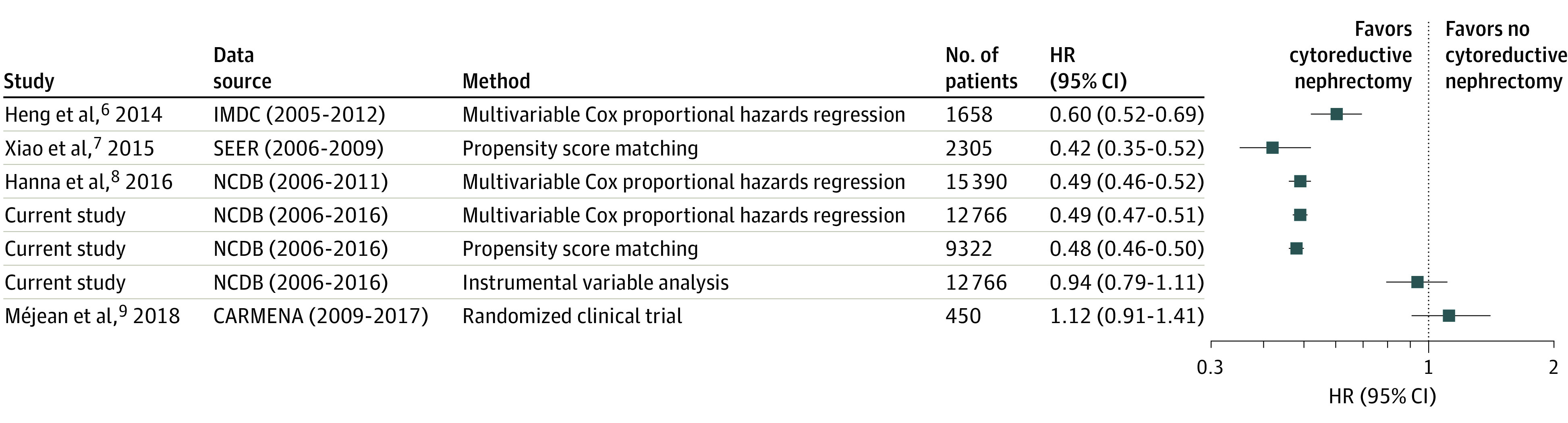
Overall Survival Outcomes for the 3 Analyses Reported in the Current Study and Relevant Published Observational Analyses and the CARMENA (Cancer du Rein Metastatique Nephrectomie et Antiangiogéniques) Randomized Clinical Trial IMDC indicates International Metastatic Renal Cell Carcinoma Database; NCDB, National Cancer Database; and SEER, Surveillance, Epidemiology, and End Results.

A forest plot was constructed using the survival outcomes generated from the 3 analyses in this study and comparing with those generated from the relevant prior observational studies as well as the CARMENA trial (Figure 2).^6,7,8,9^ The HR for OS associated with receipt of cytoreductive nephrectomy generated from the instrumental variable analysis in this study (HR, 0.92) was nearest to the HR identified in the CARMENA trial (HR, 1.12), with HRs from the analyses that used conventional adjustments falling between 0.42 and 0.60.

As a subgroup analysis, patients were stratified based on cytoreductive nephrectomy sequence with targeted therapy administration (upfront nephrectomy: 4393 patients [87.8%]; median time to nephrectomy, 23 days; IQR, 8-41 days; delayed nephrectomy: 612 patients [12.2%]; median time to nephrectomy, 120 days; IQR, 68-193 days). A forest plot summarizing OS association by cytoreductive nephrectomy sequence subgroup can be found in eFigure 4 in the Supplement.

## Discussion

Using different analytic methods to address confounding and bias in identical study populations, we found substantially different results ranging from significant to nonsignificant associations between cytoreductive nephrectomy and OS. Methods that do not adjust for unmeasured confounding identified a highly significant OS benefit for patients who underwent cytoreductive nephrectomy, whereas instrumental variable estimates did not demonstrate this association. The survival associations identified in the instrumental variable analysis more closely approximated those reported in the CARMENA trial^9^ (Figure 2). It is likely that this discrepancy reflects the fact that surgical indication for cytoreductive nephrectomy is primarily driven by factors that are not commonly measured or available in observational data sets. These findings are similar to those identified in a landmark analysis by Stukel et al,^30^ who demonstrated that instrumental variable analysis more closely approximated clinical trial outcomes than conventional adjustments for measured confounding in an observational study assessing the benefit of cardiac catheterization among patients with acute myocardial infarction.

It is worth reinforcing that instrumental variable estimates reflect the outcomes of marginal patients in the sample—those whose exposure status was primarily associated with the instrumental variable. In this instance, marginal patients are those whose cytoreductive nephrectomy status was primarily associated with their distance to the treating facility. Increasing distance to the treating facility was significantly associated with receipt of a cytoreductive nephrectomy, presumably because patients are more willing to travel to referral centers for complex surgical care with a limited number of visits, as opposed to receipt of systemic therapy that requires frequent visits for an indefinite period and can be effectively administered locally. The *F* statistic for distance to facility was 33.3, indicating adequate but relatively modest strength as an instrumental variable.^14^ The relatively modest strength of the instrumental variable decreases the marginal patient population, and therefore statistical power, compared with variables with stronger *F* statistics. However, the large patient population afforded by the NCDB (N = 12 766) was sufficient to overcome this limitation, as evidenced by an appropriately narrow 95% CI bounding the HR for cytoreductive nephrectomy status in the instrumental variable estimate (HR, 0.92; 95% CI, 0.78-1.09) (Table 3, Figure 2).

An example of power degradation associated with instrumental variable estimates can be found in a study by Macleod et al,^35^ who performed an analysis similar to the current study, but with a significantly smaller population of 537 Medicare beneficiaries, using Surveillance, Epidemiology, and End Results registry state as the instrumental variable. Despite an *F* statistic greater than 30, the marginal patient populations were inadequate to power the instrumental variable analysis, as evidenced by a very wide 95% CI for cytoreductive nephrectomy status in the instrumental variable estimate (HR, 0.29; 95% CI, 0.08-1.00). Of interest, the point estimate indicated a substantial benefit for patients who underwent cytoreductive nephrectomy, although this is difficult to interpret given the limitation regarding the marginal patient population.

Similarly, in the current study, the instrumental variable subgroup analysis of 612 patients who underwent a delayed cytoreductive nephrectomy had very wide CIs with regard to OS association (HR, 0.47; 95% CI, 0.19-1.12), suggesting significant limitations regarding the marginal patient population for this subgroup (eFigure 4 in the Supplement).

The noninferiority margin defined in the CARMENA trial was an HR of 1.20 for OS among patients who did not receive a cytoreductive nephrectomy. The instrumental variable analysis in our study reported an HR of 0.92 (95% CI, 0.78-1.09) for OS among patients who received a cytoreductive nephrectomy (Table 3, Figure 2). After inverting the β-coefficient signs, the HR for OS is 1.09 (95% CI, 0.92-1.28) for patients who did not receive a cytoreductive nephrectomy. Note that the upper bound of the 95% CI falls beyond the noninferiority margin declared in the CARMENA trial, indicating that a minimal clinically important difference cannot be ruled out with 95% probability using this definition.

### Limitations

This analysis has several limitations. Primarily, as noted in the Discussion section, instrumental variable analyses functionally compare marginal patient populations within the overall cohort, potentially limiting the generalizability of the results. Any retrospective analysis of observational data is subject to selection bias from unmeasured confounding, although the multivariable Cox proportional hazards regression and propensity score–matched analyses presented in this study are exposed to this bias to a substantially greater degree than the instrumental variable analysis. This analysis was not designed to correct for the immortal time bias introduced via treatment stratification. The NCDB broadly categorizes systemic therapies such that the names of the medications are not available. Likewise, specific information is lacking regarding subsequent lines of therapy beyond the first-line treatment. The NCDB does not include data on kidney function, body mass index, performance status, volume and site of metastatic disease, International Metastatic Renal Cell Carcinoma Database risk category, treatment-related toxic effects, response rates, progression-free survival, or recurrence-free survival, all of which would have contributed to this analysis if available for study. Finally, most current guideline–recommended first-line management regimens for metastatic ccRCC use immune checkpoint blockade as opposed to targeted therapy monotherapy, potentially limiting the applicability of these data. Randomized clinical trials will be necessary to assess the role of cytoreductive nephrectomy in combination with immune checkpoint blockade.

## Conclusions

Consistent with results from the CARMENA trial, in this cohort study, instrumental variable estimates did not demonstrate a survival advantage associated with cytoreductive nephrectomy for patients with metastatic ccRCC. Methods that do not adjust for unmeasured confounding, multivariable Cox proportional hazards regression, and propensity score matching demonstrated a substantial benefit associated with cytoreductive nephrectomy. This discrepancy likely reflects the fact that surgical indication for cytoreductive nephrectomy is primarily driven by factors that are not commonly measured or available in observational data sets.

## Notes

Supplement. **eFigure 1.** Kaplan-Meier Distributions for Overall Survival **eFigure 2.** Propensity Score Matching **eFigure 3.** Assumptions Underlying Instrumental Variable Analysis, Distance to Facility, and F Statistics **eFigure 4.** Forest Plot Visualizing Overall Survival Outcomes for the Three Analyses Reported in the Present Study, Further Stratified Into Subgroups Based on Up-Front Versus Delayed Cytoreductive Nephrectomy (CN) **eTable 1.** Baseline Patient and Tumor Characteristics, Stratified by Cytoreductive Nephrectomy Status, in the Post-Matched Cohort (N = 9,322) **eTable 2.** Multivariable Cox Regression for Overall Survival, Including as Covariates Variables From the Post-Matching Univariable Analysis With *P* Values <0.10, in Addition to Cytoreductive Nephrectomy Status **eTable 3.** Baseline Patient and Tumor Characteristics, Stratified by Distance to Facility Tertiles
