# Error in Figure 2

**DOI:** 10.1001/jamanetworkopen.2022.20420

**Published:** 2022-06-15

In the Original Investigation titled “Survival Outcomes Associated With Cytoreductive Nephrectomy in Patients With Metastatic Clear Cell Renal Cell Carcinoma,”^1^ published May 16, 2022, in Figure 2, the wrong HR and 95% CI values were reported for the instrumental variables analysis outcome. The correct values are 0.92 (95% CI, 0.78-1.09). This article has been corrected.^1^
