# Supplementary material for: Survival Outcomes Associated With Cytoreductive Nephrectomy in Patients With Metastatic Clear Cell Renal Cell Carcinoma
Source: JAMA Netw Open. 2022 May 16;5(5):e2212347. doi: 10.1001/jamanetworkopen.2022.12347 (PMC9112069; doi:10.1001/jamanetworkopen.2022.12347)
Supplement: Supplement. — eFigure 1. Kaplan-Meier Distributions for Overall Survival eFigure 2. Propensity Score Matching eFigure 3. Assumptions Underlying Instrumental Variable Analysis, Distance to Facility, and F Statistics eFigure 4. Forest Plot Visualizing Overall Survival Outcomes for the Three Analyses Reported in the Present Study, Further Stratified Into Subgroups Based on Up-Front Versus Delayed Cytoreductive Nephrectomy (CN) eTable 1. Baseline Patient and Tumor Characteristics, Stratified by Cytoreductive Nephrectomy Status, in the Post-Matched Cohort (N = 9,322) eTable 2. Multivariable Cox Regression for Overall Survival, Including as Covariates Variables From the Post-Matching Univariable Analysis With P Values <0.10, in Addition to Cytoreductive Nephrectomy Status eTable 3. Baseline Patient and Tumor Characteristics, Stratified by Distance to Facility Tertiles [file jamanetwopen-e2212347-s001.pdf]

Supplementary Online Content

Chakiryan NH, Gore LR, Reich RR, et al. Survival outcomes associated with cytoreductive nephrectomy in patients with metastatic clear cell renal cell carcinoma. *JAMA Netw Open*. 2022;5(5):e2212347. doi:10.1001/jamanetworkopen.2022.12347

eFigure 1. Kaplan-Meier Distributions for Overall Survival

eFigure 2. Propensity Score Matching

eFigure 3. Assumptions Underlying Instrumental Variable Analysis, Distance to Facility, and F Statistics

eFigure 4. Forest Plot Visualizing Overall Survival Outcomes for the Three Analyses Reported in the Present Study, Further Stratified Into Subgroups Based on Up-Front Versus Delayed Cytoreductive Nephrectomy (CN)

eTable 1. Baseline Patient and Tumor Characteristics, Stratified by Cytoreductive Nephrectomy Status, in the Post-Matched Cohort (N = 9,322)

eTable 2. Multivariable Cox Regression for Overall Survival, Including as Covariates Variables From the Post-Matching Univariable Analysis With *P* Values < 0.10, in Addition to Cytoreductive Nephrectomy Status

eTable 3. Baseline Patient and Tumor Characteristics, Stratified by Distance to Facility Tertiles

This supplementary material has been provided by the authors to give readers additional information about their work.

© 2022 Chakiryan NH et al. *JAMA Network Open.*

eFigure 1. Kaplan-Meier Distributions for Overall Survival

A: Entire cohort (N = 12,766). B: Post-matching cohort (N = 9,322).

A

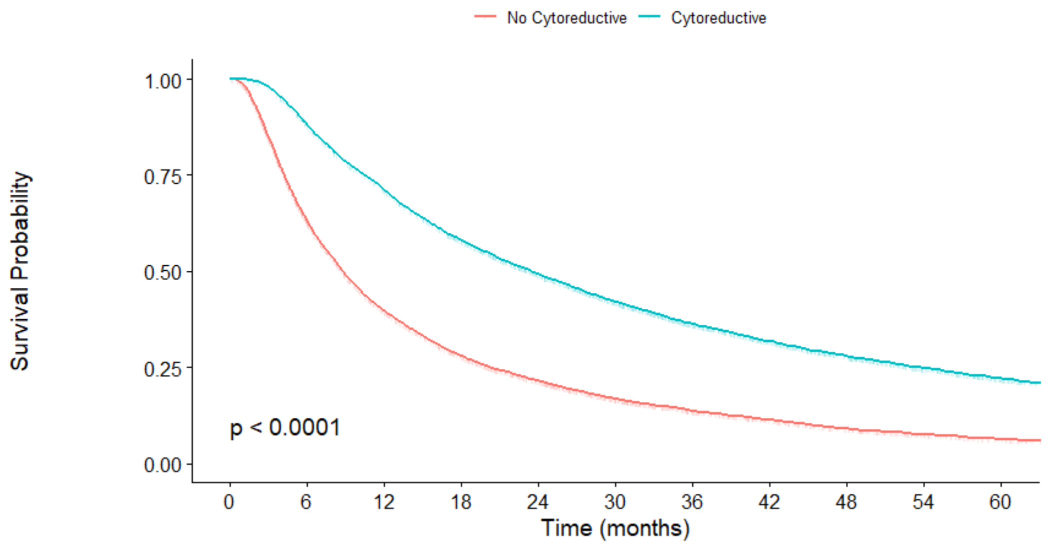

Number at risk

Time (months)	0	6	12	18	24	30	36	42	48	54	60
No Cytoreductive	7761	4780	2935	2016	1461	1035	755	556	399	275	198
Cytoreductive	5005	4389	3517	2826	2296	1845	1446	1149	917	717	558

B

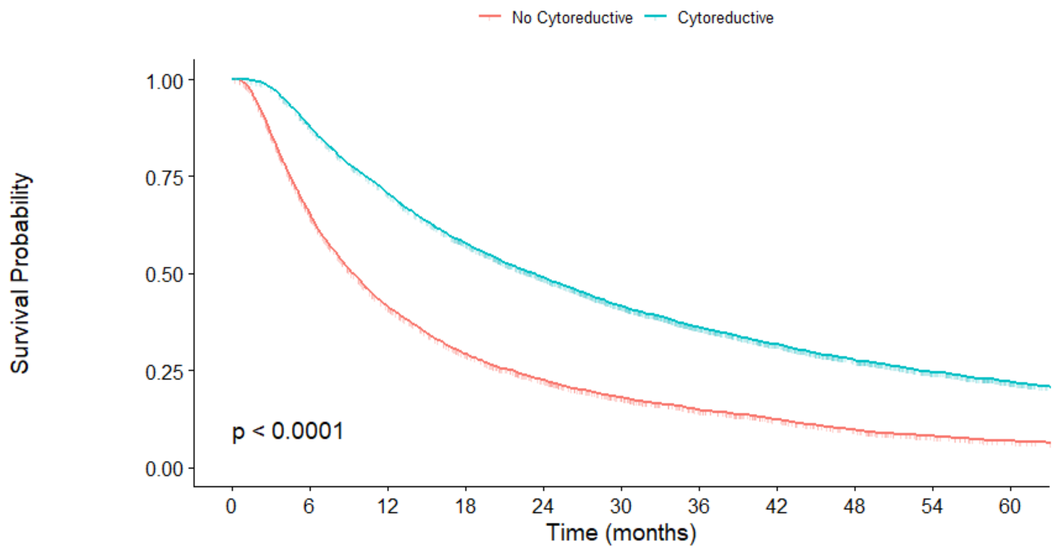

Number at risk

Time (months)	0	6	12	18	24	30	36	42	48	54	60
No Cytoreductive	4661	2979	1849	1267	929	681	507	377	263	181	131
Cytoreductive	4661	4072	3250	2614	2116	1690	1327	1055	836	653	506

© 2022 Chakiryan NH et al. *JAMA Network Open.*

eFigure 2. Propensity Score Matching

A: Propensity score distributions before and after propensity matching (note: the y-axis scaling differs between graphs). "Treatment" units represent patients in the cytoreductive nephrectomy group, and "control" units represent patients who did not undergo a cytoreductive nephrectomy. B: Jitter-plot demonstrating patients who were matched and dropped during the propensity score matching process.

A

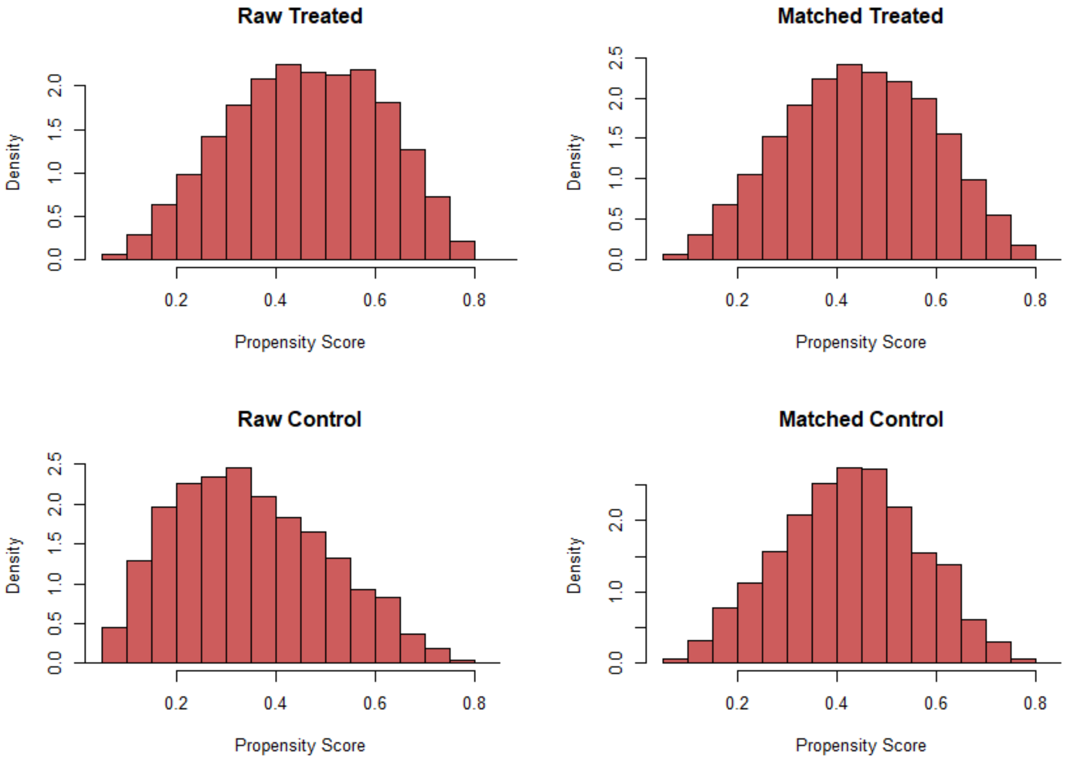

B

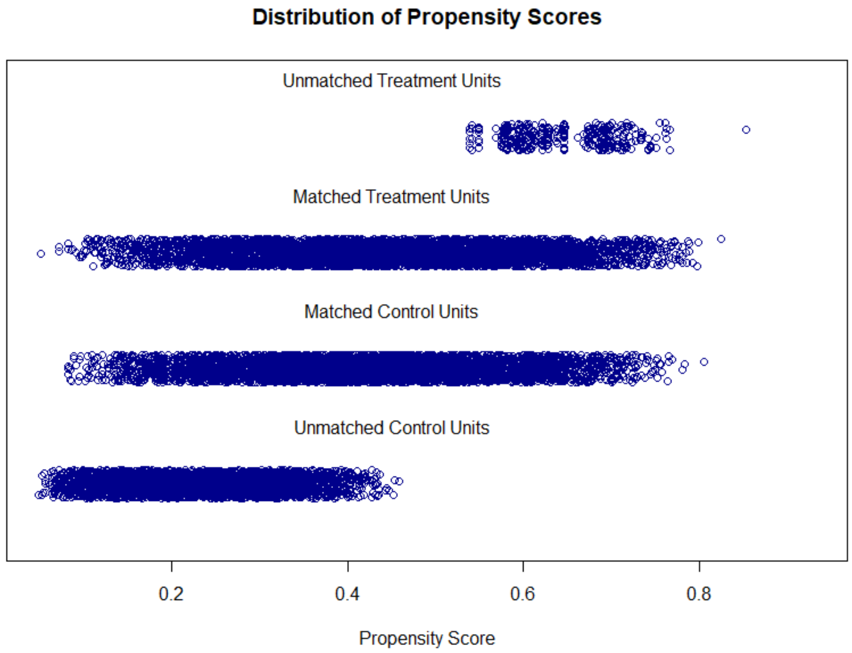

© 2022 Chakiryan NH et al. *JAMA Network Open.*

eFigure 3. Assumptions Underlying Instrumental Variable Analysis, Distance to Facility, and F Statistics

A: Directed acyclic graph of the assumptions underlying instrumental variable analysis (Z = instrumental variable, X = exposure variable, T = outcome, L = measured confounding variables, U = unmeasured confounding variables, arrows indicate causal associations). B: Histogram depicting the distribution of patients by distance to facility in ln(miles); blue line indicates the proportion of patients undergoing a cytoreductive nephrectomy by distance to facility. C: F-statistics with their associated P values evaluating distance to facility as an instrumental variable, assessing the first-stage model with and without distance to facility as a covariate, and a multivariable Cox regression for OS with and without distance to facility as a covariate.

A

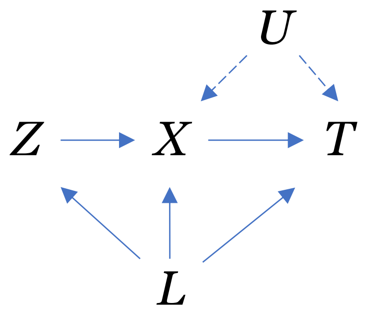

B

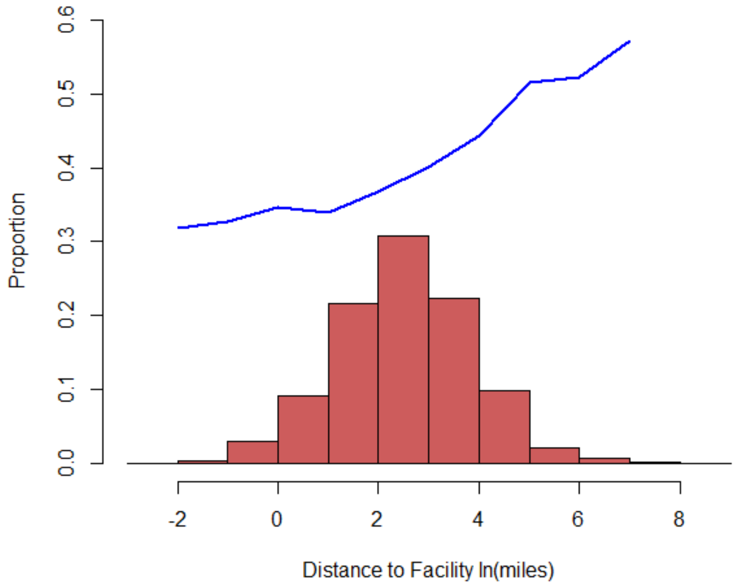

C

	F Statistic	P
**First-Stage (Multivariable Logistic Regression for CN status)**		
Without Distance to Facility		
With Distance to Facility	33.3	< 0.001
**Multivariable Cox Regression for OS**		
Without Distance to Facility		
With Distance to Facility	1.7	0.20
**Multivariable models include covariates: Age, Sex, Race, Charlson, Facility Type, Year of Diagnosis, cT, cN, +/- Distance to fac.*		

© 2022 Chakiryan NH et al. *JAMA Network Open.*

eFigure 4. Forest Plot Visualizing Overall Survival Outcomes for the Three Analyses Reported in the Present Study, Further Stratified Into Subgroups Based on Up-Front Versus Delayed Cytoreductive Nephrectomy (CN)

**Methodology**	**Subgroup**	**N with CN**	**HR [95% CI]**
Multivariable Cox Regression			
	Any CN	5,005	0.49 [0.47-0.51]
	Up-front CN	4,393	0.50 [0.48-0.52]
	Delayed CN	612	0.39 [0.36-0.43]
Propensity Score Matching			
	Any CN	5,005	0.48 [0.46-0.50]
	Up-front CN	4,393	0.49 [0.47-0.52]
	Delayed CN	612	0.39 [0.35-0.43]
Instrumental Variable Analysis			
	Any CN	5,005	0.92 [0.78-1.09]
	Up-front CN	4,393	0.97 [0.81-1.16]
	Delayed CN	612	0.47 [0.19-1.12]

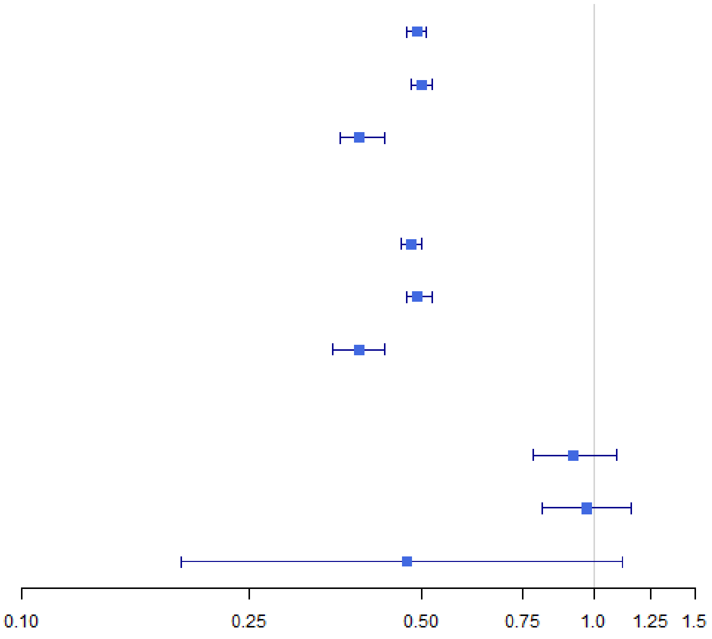

© 2022 Chakiryan NH et al. *JAMA Network Open.*

eTable 1. Baseline Patient and Tumor Characteristics, Stratified by Cytoreductive Nephrectomy Status, in the Post-Matched Cohort (N = 9,322)

Characteristic	No Cytoreductive, N = 4661[1]	Cytoreductive, N = 4661[1]	p-value[2]
**Age**	62 (54, 69)	61 (54, 68)	0.055
**Sex**			0.8
Male	3190 (68%)	3180 (68%)	
Female	1471 (32%)	1481 (32%)	
**Race**			0.1
White	4135 (89%)	4172 (90%)	
Black	331 (7.1%)	281 (6.0%)	
Other	195 (4.2%)	208 (4.5%)	
**Charlson-Deyo**			0.8
0	3322 (71%)	3350 (72%)	
1	990 (21%)	983 (21%)	
2	235 (5.0%)	223 (4.8%)	
3+	114 (2.4%)	105 (2.3%)	
**Facility Type**			0.2
Academic	1877 (40%)	1942 (42%)	
Non-Academic	2784 (60%)	2719 (58%)	
**Year of Diagnosis**	2012 (2010, 2014)	2012 (2010, 2014)	0.5
**cT**			0.03
cT1	959 (21%)	881 (19%)	
cT2	1630 (35%)	1606 (34%)	
cT3	1699 (36%)	1826 (39%)	
cT4	373 (8.0%)	348 (7.5%)	
**cN**			0.005
cN0	3123 (67%)	3249 (70%)	
cN+	1538 (33%)	1412 (30%)	

[1] Statistics presented: median (IQR); n (%)

[2] Statistical tests performed: Wilcoxon rank-sum test; chi-square test of independence

© 2022 Chakiryan NH et al. *JAMA Network Open.*

eTable 2. Multivariable Cox Regression for Overall Survival, Including as Covariates Variables From the Post-Matching Univariable Analysis With *P* Values < 0.10, in Addition to Cytoreductive Nephrectomy Status

**Characteristic**	**HR**[1]	**95% CI**[1]	**p-value**
**Age (per year)**	1.00	1.00, 1.00	0.11
**cT**			
cT1	—	—	
cT2	1.19	1.11, 1.27	<0.001
cT3	1.22	1.14, 1.30	<0.001
cT4	1.32	1.20, 1.46	<0.001
**cN**			
cN0	—	—	
cN+	1.40	1.33, 1.47	<0.001
**Cytoreductive Nephrectomy**	0.48	0.46, 0.50	<0.001

[1] HR = Hazard Ratio, CI = Confidence Interval

© 2022 Chakiryan NH et al. *JAMA Network Open.*

eTable 3. Baseline Patient and Tumor Characteristics, Stratified by Distance to Facility Tertiles

	Distance to Facility Tertiles			
**Characteristic**	**T1**, N = 4303[1]	**T2**, N = 4211[1]	**T3**, N = 4252[1]	**p-value**[2]
**Age**	64 (56, 72)	63 (56, 71)	62 (55, 69)	<0.001
**Sex**				<0.001
Male	2855 (66%)	2912 (69%)	2977 (70%)	
Female	1448 (34%)	1299 (31%)	1275 (30%)	
**Race**				<0.001
White	3616 (84%)	3757 (89%)	3833 (90%)	
Black	469 (11%)	301 (7.1%)	263 (6.2%)	
Other	218 (5.1%)	153 (3.6%)	156 (3.7%)	
**Charlson-Deyo**				0.003
0	2959 (69%)	2981 (71%)	3059 (72%)	
1	925 (21%)	910 (22%)	844 (20%)	
2	286 (6.6%)	213 (5.1%)	241 (5.7%)	
3+	133 (3.1%)	107 (2.5%)	108 (2.5%)	
**Facility Type**				<0.001
Academic	1135 (26%)	1511 (36%)	2267 (53%)	
Non-Academic	3168 (74%)	2700 (64%)	1985 (47%)	
**Year of Diagnosis**	2012 (2009, 2014)	2012 (2010, 2014)	2012 (2010, 2015)	<0.001
**cT**				0.047
cT1	1121 (26%)	1055 (25%)	1006 (24%)	
cT2	1313 (31%)	1336 (32%)	1285 (30%)	
cT3	1368 (32%)	1326 (31%)	1456 (34%)	
cT4	501 (12%)	494 (12%)	505 (12%)	
**cN**				0.3
cN0	2647 (62%)	2657 (63%)	2641 (62%)	
cN+	1656 (38%)	1554 (37%)	1611 (38%)	
**Cytoreductive Nephrectomy**	1492 (35%)	1624 (39%)	1889 (44%)	<0.001

[1] Statistics presented: median (IQR); n (%)

[2] Statistical tests performed: Kruskal-Wallis test; chi-square test of independence

© 2022 Chakiryan NH et al. *JAMA Network Open.*
